# Growing up with a sibling with depression: A qualitative study in Israel

**DOI:** 10.1371/journal.pone.0290999

**Published:** 2023-08-31

**Authors:** Inbar Levkovich, Michal Labes

**Affiliations:** Faculty of Graduate Studies, Oranim Academic College, Kiryat Tiv’on, Israel; University of Environment and Sustainable Development, GHANA

## Abstract

Depression has major consequences for the entire family, among them emotional distress, disrupted daily routine and social damage caused by negative stigmas. The aim of this study was to explore the retrospective experiences of young adults who lived with a sibling with depression while they were adolescents. The present study adopted a qualitative-phenomenological approach. The research participants were recruited via purposive sampling on social networks across Israel from May to September 2022. Semi-structured interviews were conducted with 21 participants aged 18–29 who lived with a sibling with depression during their adolescence. Data collection continued until saturation of concepts was reached. The results underwent thematic analysis. Three themes emerged from the qualitative analyses: 1) “I felt like I was living in a minefield”: Adolescence while living with a sibling with depression; 2) “One step forward and two steps back”: Siblings’ coping strategies; 3) “My parents were not there for me when I needed them”: Participants’ experiences with their parents during their adolescence. The research findings indicate that adolescents who grew up with a sibling affected by depression had to cope with an acute family crisis, whose serious ramifications required emotional and social support. Mental health professionals and counselors working within educational institutions and the broader community should provide support and intervention for adolescents who have siblings struggling with depression. This intervention may take the form of individual or group therapy that aims to foster a sense of belonging and help affected individuals. Creating a supportive environment that meets the needs of the affected siblings is also crucial in addressing this issue effectively.

## Introduction

Depression is a common and serious psychiatric disorder that finds expression in depressed mood, loss of pleasure and interest, feelings of emptiness, sleep disruptions, and loss of appetite [1]. Depression is the most widespread psychiatric disorder among adolescents, with a prevalence of 13.3% - 25% [2–4]. Based on a nationwide representative sample of Israeli adolescents between the ages of 14 and 17, the Israeli Survey of Mental Health among Adolescents (ISMEHA) determined a depression prevalence rate of 14.6% [5]. Depression has consequences for the emotional, social, academic, and developmental condition of affected individuals and for their future [6, 7].

Not only does depression affect the individual sufferer. It also affects the entire family. Young adults who lived with a sibling with depression are themselves at increased risk for distress and mental illness [8–10]. Research shows that the nature of the sibling relationship and the degree to which siblings provide one another social support are negatively associated with the appearance of depressive symptoms in both siblings [6, 11]. To better understand the experience of adolescents who live in the same house as a sibling with depression, it is necessary to understand the sibling and family relationships.

Living in the same house as a sibling with depression is liable to have a negative impact on an individual’s emotional well-being, produce feelings of distress and anxiety, and cause the individual to perceive of the sibling as a burden [8–10]. The results of a study that examined the experiences of adolescents living with a sibling diagnosed with mental illness pointed to feelings of being rejected by the environment, a sense of having lost one’s social standing, verbal abuse and harassment from peers and neighbors, feelings of lack of self-worth, and negative stigmas toward the family [12]. On the other hand, adolescents living with a sibling with a mental disorder can also develop high levels of compassion and empathy [10].

Adolescents living with a sibling with depression are themselves at high risk of developing depression. There are several explanations for this, including genetics, similar life experiences, parental resources devoted to caring for the sibling with depression, and family climate [4]. Barnett and Hunter [8] found that 19% of these siblings showed signs of depression and anxiety, 16% displayed aggressive behavior, and 12% exhibited defiant rebellious behavior.

Despite its resemblance to adolescence, the period of emerging adulthood is marked by unique features that define it as a separate developmental state. Unlike adolescents, young adults have already reached physical and sexual maturity. They are legally responsible for their actions and can therefore decide what to do, where to work, and where to live. Yet unlike mature adults, they are not yet stable financially, personally, or professionally [13]. Several studies indicate that the experiences of young adults during the period of emerging adulthood are related to and influenced by their behavior during adolescence [14–17].

Many studies have examined the experiences of adolescents with depression and the perspectives of their parents [18, 19]. Yet empirical knowledge is lacking regarding the experiences of their siblings. In addition, previous studies examined a variety of mental illnesses [20, 21], whereas the current study seeks to focus exclusively on depression. The objective of this study was to enhance our understanding of the adolescent experience of young adults who grew up together with a sibling with depression. The research examined the following question: What is the nature of the retrospective experiences of participants who lived with a sibling with depression while they were adolescents?

## Materials and methods

The study was conducted according to the qualitative phenomenological approach, which attempts to obtain an in-depth understanding of the studied phenomenon by entering the world and experiences of the participants [22]. The approach uses descriptions provided by the study participants and their reflections on phenomena to identify central experiences [23]. Guidelines for ensuring rigor and reflexivity in qualitative research were followed and the researchers completed the COREQ checklist for reporting qualitative data [24].

### Participants

Twenty-one young adults who lived with a sibling with depression while they were adolescents participated in the study. The participants, who ranged in age from 18–29 years old, all grew up in the same house as a sibling with depression (age, M  =  22.39, SD = 3.47; gender, 57.14% female). Most of the participants were single (90.47%) and none had children of their own. Inclusion criteria were: 1) lived in the same house with a brother or sister with depression when they were adolescents; 2) between the ages of 18 and 29 years old; 3) lived at home during adolescence (not at boarding schools); 4) biological siblings from the same two parents (e.g., not siblings living in foster care). These inclusion criteria were used to create a homogeneous group and reduce intervening variables. Exclusion criteria were: 1) brother or sister with a history of psychosis; 2) participants currently exhibiting suicidal intent or severe depression because of concerns about the adverse effects of the interview process; 3) participants who identified themselves as having depression (Table 1).

**Table 1:** Sample characteristics (N = 21).

Characteristic	N	%
Sibling’s age* *M* (SD)	22.39 (3.74)	
Sibling’s gender		
Male	9	42.86
Female	12	57.14
Sibling’s marital status		
Single	19	90.47
Married	2	9.53
No. of children in the family M (SD)	3.72 (2.24)	
Current age of sibling with depression M (SD)	22.41 (5.07)	
Parents’ current marital status		
Divorced or separated	7	33.34
Married	14	66.66

*Current age of research participant

### Procedure

The research participants were recruited via purposive sampling on social networks (e.g., Facebook) across Israel. Young adults who grew up with siblings with depression were examined. Participants responded to a letter posted on social media by sending an email with their details. After receiving a comprehensive explanation about the general research aims, interviewees signed an informed consent form. For the interview, they also were required to submit a medical record documenting their sibling’s history of depression during their own period of adolescence. Three participants who did not submit a medical document testifying to their sibling’s depression were eliminated from the study.

The interviewers (IL and ML) were both females. IL is a psychotherapist (PhD) and ML holds a master’s degree in educational counselling. Both are experienced in conducting qualitative research. Prior to conducting the interviews, the interviewers reflected on the identities, social locations, assumptions, and life experiences they brought to the research endeavor and contemplated their interactions with the interviewees. The interviews took place between May and September 2022 and were conducted via the Zoom platform to minimize personal contact during the COVID-19 pandemic. The interviews lasted about one hour, on average.

All interviews were recorded and transcribed verbatim. The interviews were conducted in Hebrew, and the transcripts were translated into English. Each translation was verified by two native speakers, one of whom is a professional translator. Once theoretical saturation was reached (i.e., additional interviews yielded no new material for analysis), no additional interviews were conducted.

### Research tools

The qualitative data in this study were collected by means of in-depth, semi-structured interviews. The interviews were conducted based on an interview guide that included significant key areas but were flexible enough to allow a dialogue to develop between the interviewer and the interviewee, as well as to facilitate meaningful self-expression [23] (Table 2).

**Table 2:** Interview questions posed to participant who grew up with sibling with depression.

Questions
• What does depression mean to you?
• Describe your sibling’s experience with depression (when did it begin, when was it diagnosed, what changes occurred over the years, treatments, additional diagnoses).
• What difficulties did you experience during your adolescence while living with a sibling with depression?
• What helped advance your relationship with your sibling?
• What changes took place in your relationship over the years?
• Is your sibling still coping with depression today?
• Tell me about your relationship with your parents. Did your relationship change over the years, and if so, how? Before and after your sibling’s depression began?
• Describe the relationship between your parents and your sibling? In your opinion, did your sibling’s depression affect your parents (emotional state, relations between them, occupational situation)?
• What did you need from your parents during your adolescence? What support did you receive? What did you need from them that you felt you did not receive?
• Describe the relations in your nuclear family during your adolescence.

### Data analysis

The data collected in the interviews were analyzed thematically in six phenomenological phases [25]. In Phase 1, the researchers read and reread the transcribed data to become familiar with it. Phase 2 involved generating a list of initial ideas, features, and repeated patterns and then forming initial codes by systematically coding and collating features of the data relevant to the research aim. In Phase 3, the coded information was collated and a thematic map was generated to express the essence of the phenomena. The codes were then combined into potential themes that reflected major features and patterns in the data. Phase 4 involved identifying themes. In Phase 5, the data were rechecked against the original transcripts and audio recordings. Phase six entailed describing the themes and the underlying story describing how the phenomenon was experienced and adding illustrative quotations [25].

### Ethical considerations

This study was approved by the Ethical Review Board of the Oranim College Research Ethics Committee, Israel) No. 117/2022) and conformed to the Declaration of Helsinki [26]. Informed consent was obtained from all participants, who were also made aware that any publications resulting from the study would not include identifying information. To maintain confidentiality, the research team clarified at the outset of the interviews that no names or identifying information would be published, and this was reiterated in the written informed consent form the participants signed. In this manuscript, participants are referred to by assigned pseudonyms or referred to as “the participants”.

## Results

Analysis of the interviews yielded three themes that shed light on the experiences of the research participants as adolescents growing up with a sibling with depression (Table 3).

**Table 3:** Classification of main theme and subcategories.

Main Theme	Subcategories
**“I felt like I was living in a minefield”**	• Emotional difficulties emerging when the sibling was diagnosed. • Changes in the sibling relationship while dealing with the diagnosed sibling. • Participants experienced difficulties as adolescents but were forced to repress them due to the central place of the sibling’s depression. • Constant tension and a stormy atmosphere at home. • Participants witnessed behaviors such as self-harm, verbal and physical violence directed towards them or their parents
**"One step forward and two steps back”:** Siblings’ **coping strategies**	• Ways of coping with the new situation. • Many felt they had to help, even at the price of self-nullification. • Others reported that their sibling’s problems caused them fear and avoidance.
**“My parents were not there for me when I needed them”**	• Changes in relations with parents as a result of the sibling’s depression. • Assuming parental roles to make it easier for parents to cope with sibling’s depression. • Sense of being distanced from and cut off from the parents because they were not available.

### Theme 1: “I felt like I was living in a minefield”: Adolescence while living with a sibling with depression

The research participants described the period before their sibling was diagnosed with depression as “foggy”. During this period, they felt a lack of clarity and confusion regarding their sibling’s condition. Some of the participants mentioned their difficulty in understanding their sibling’s emotional distress. Often, they accused their sibling of disrespecting their parents, acting like a crybaby, exaggerating things or trying to attract attention. Others felt that their sibling was suffering but they had no way to help. The research participants described their complicated feelings during that period, particularly their anger, frustration, helplessness, and loneliness. After the depression diagnosis, the participants stated they were able to identify depressive symptoms in their sibling, such as social problems, diminished sharing and fewer emotional discussions, a tendency toward seclusion at home, suicidal thoughts and self-harm, profound sadness or loss of meaning in life. In most cases, the depressive symptoms identified after the diagnosis worsened with time, and the research participants’ sense of distress increased accordingly.

When my sister came home from the army, all she did was cry, repeatedly state how bad things were for her, tell us that the other girls in the course did this or that to her and she couldn’t find any friends there…. For me this was just that she is a drama queen, she exaggerates everything, that’s how it started… at some point I really began to be angry with her because it was impossible to talk to her (female participant, 29 years old).

Some of the participants felt that their sibling’s illness was an existential state of emergency that generated a storm at home. Participants described a situation in which they, as adolescents, experienced ups and downs in their lives but were forced to repress these due to the centrality of their sibling’s illness. Batia, 20 years old, describes her frustration because her brother’s pain did not leave any room for the rest of the family to cope with their problems. Indeed, a hierarchy of challenges emerged, and she felt she was at the bottom of the ladder:

I felt like I was living in the middle of a huge minefield. I remember times when I felt I was not allowed to get angry, not allowed to feel insulted, not allowed to feel anything, because other people have things so much worse. I remember when my brother broke up with his last serious partner, and he just didn’t come home, and it was as if…. no one else was allowed to have any problems in life because his situation was so difficult (female participant, 20 years old).

Within the storm and huge family crisis, many of the participants felt they had to remain constantly on alert. They felt they had to enlist themselves to help with the family crisis, even at the price of self-sacrifice and self-nullification. These participants put aside their personal commitments and desires, including social connections, hobbies, or attention from their parents. Doing this exacted a steep emotional price from them, and they experienced negative emotions such as anxiety, stress, and sadness. Yet nevertheless, the research participants felt they had no choice but to do their part.

When my sister was receiving outpatient care, she was at home all the time and needed supervision all the time. Someone always had to be with her and keep her occupied. On many occasions she would lock herself in the bathroom or in her room and my parents would cry … that was always so chaotic, with everyone crying and screaming because they were convinced she would never come out. As the person who was closest in age to her, I was always the one who had to deal with her, take her places, stay with her (male participant, 29 years old).

A significant number of research participants witnessed behaviors such as self-harm, verbal and physical violence directed towards them or their parents, and hospitalization in psychiatric wards. These situations led to intense fear, worry, stress, anxiety, instability, and insecurity within their own homes. The research participants did not know how to handle the reactions of their siblings, who were required to perform various actions even when they were struggling with depression. The unexpected responses surprised the research participants, causing them panic and shock.

During a youth movement trip, I began telling my sister how I felt about her condition, but she showed me her cuts, silencing me. I felt hurt and scared by her actions because this was the first time I had witnessed her self-harming behaviour. I was upset but tried to reassure her by asking her whether she would hurt herself again (male participant, 23 years old).

Participants in the study viewed their sibling’s illness as an existential crisis, creating turmoil at home and resulting in feelings of emotional distress, frustration, and loneliness. They described their adolescence as a time of suppressed emotions due to the importance of their sibling’s illness. Amidst this family turbulence, many felt a constant need to be on alert and were willing to put the family’s needs above their own, even at the cost of personal sacrifice.

### Theme 2:"One step forward and two steps back”: Siblings’ coping strategies

In the midst of the storm and the great rift, many of the siblings felt they needed to remain constantly vigilant and ready to act. They felt they had to mobilize themselves to help the family, even at the cost of sacrificing their personal needs. These participants reported that their sibling’s illness "came at their expense”. They described pushing aside personal obligations and wishes, including social relationships, hobbies, or attention from their parents. Their sibling’s condition exacted many personal costs from them, including damaging their social relationships and eliciting negative feelings such as sadness, stress, pressure, and anger. Still, the research participants felt they had no choice but to carry out their obligations to the family.

During the periods my sister was treated as an outpatient, she was always at home and always had to be supervised. I always had to be with her and keep her occupied. On many occasions she would lock herself up, lock herself in the shower, or lock herself in the room and my parents would cry…. Because I’m the closest to her in age, I tried to be with her because she always really trusted me and was very attached to me. So I always kept her occupied and stayed with her. Many times I would take her to the beach or I would just stay with her at home, trying to find things we had in common (female participant, 19 years old).

The participants stated that their relationship with their siblings during that period was not reciprocal but rather focused on the sibling with depression. They took care of their sibling, devoted emotional resources and time, tried to engage the sibling in conversation, and showed concern for or physically protected their sibling. In contrast, the affected sibling was engrossed in his/her own world and was not emotionally available to pay attention to what was happening in the world of the research participants:

I was totally there. I was fully engrossed, in action mode. I didn’t ask too many questions. Apparently I understood there really wasn’t anyone to talk to. It was an emergency situation… I often sat beside her all night long, I engaged in guided imagery, lots of discussions, I was totally drawn in and devoted myself totally, and afterwards I was totally drained (female participant, 22 years old).

Other participants, in contrast, reported that their sibling’s difficulties frightened them to the point of avoidance. As young adults they recognized their sibling’s condition made them feel unwanted, rejected and sometimes even in the way at home. They expressed fears that society would assign a negative label to them because of their sibling’s illness. Hence they spent many hours at the homes of friends and relatives and devoted lots of time to demanding sports activities or hobbies. They defined their relationship with their sibling as “nonexistent”, possibly due to repression resulting from having trouble seeing their sibling in this situation. Despite the lack of communication between the siblings, the research participants reported feelings of guilt, anger, fear, helplessness, and distress when thinking about their sibling.

At home they saw me as a guest, someone who comes home on weekends and holidays. I also wasn’t so involved in what was going on at home, certainly not in the lives of my siblings. As a child it was very difficult to communicate with my sibling on a certain level, I didn’t have the tools for that (male participant, 25 years old).

Many participants felt the need to prioritize their family’s well-being over their own personal needs, sacrificing social relationships and hobbies. The illness of their sibling often led to strained social connections and feelings of sorrow and stress. Nevertheless, they saw their responsibilities towards their family as inescapable. On the other hand, some participants reacted to their sibling’s challenges with fear and avoidance. Internal feelings of rejection and concerns about societal stigmatization drove them to find solace with friends or immerse themselves in intense hobbies.

### Theme 3: “My parents were not there for me when I needed them”: Participants’ experiences with their parents during their adolescence

The young adult research participants reported that when they were adolescents, their sibling’s depression was a factor in how close they felt to their parents, whether they were able to share experiences with their parents, and whether they could rely on their parents for support. Some of the participants described how they lost the positive and significant relationship they had with their parents before their sibling’s illness. Nili, age 28, reported that she had a good relationship with her parents before the onset of her sister’s illness. Yet after her sister began experiencing symptoms of depression, Nili said that her parents focused all their energy on her sister, leaving her feeling neglected. She recalled that “they no longer seemed really interested in me”.

According to Ido, before the onset of his sister’s depression he had many problems in elementary school and relied on his parents to help him solve them. When his sister’s depression began to emerge and as it escalated, Ido stopped “causing problems” or asking for his parents’ help. In retrospect he described himself during high school as “a remote and easy kid that my parents did not need to deal with very much”. The feeling that the parents are not available or attentive to their needs came up in other interviews as well. For most of the participants, their relationship with their parents was marked by feelings of rejection, disappointment, and abandonment. Most noted that they yearned for a deeper, more inclusive, and more meaningful relationship with their parents. The research participants did not want to disappoint or make things any harder for their parents, who already were so sad and in so much pain, so they tried to please them and win their love. They tried to be “perfect children” and to avoid behavior or situations that might make their parents uncomfortable. Sarah commented on the need to meet her parents’ expectations and maintain an external image of being problem-free, even though inside she was struggling with many difficulties. She behaved this way to satisfy her parents:

I excel and am wonderful and do everything I should, so that on the outside there is no reason to be ashamed of me, as opposed to… A brother with depression is something to hide, but about me they can brag all the time (female participant, 24 years old).

Some of the participants reported that they tried to assume parental roles at home, such as watching their sibling, taking him/her to treatments, or doing all sorts of chores to make things easier for their parents. This desire to please their parents was typical of many of the research participants and caused them fatigue and burnout:

On the weekends I would come home from university and spend lots of time there doing things my mother never had time for, like cleaning the house, taking the dog for a walk, doing laundry, anything that my mother could never find time for because she was busy with my sister 24/4 (female participant, 20 years old).

Participants also had to cope with the challenge posed by their parents’ request that they conceal their sibling’s condition from people outside the family. Sometimes the parents directly asked them “to hide”, while in other cases the participants extrapolated this from their parents’ behavior. Hence, the participants were left alone to cope with difficult incidents and complex experiences, without being allowed to share their feelings and receive support from those around them. For many this led to isolation and sadness.

They always told me not to tell anyone. I remember that my mother insisted that I keep this a secret, and also my father… It was also clear to me from their behavior and their language that this is what was expected of me. Today I understand how much this lack of communication, these scare tactics, influenced me (male participant, 22 years old).

Many participants noted a deterioration in their positive relationships with their parents after their sibling’s illness. They felt rejected, disappointed, and abandoned. Many expressed a longing for a deeper connection with their parents and aimed to be the ’perfect child’ to please them, even amidst feelings of neglect. Additionally, they faced the challenge of keeping their sibling’s condition a secret, as requested by their parents.

## Discussion

The effects of depression are not limited to the afflicted individual, for they also extend to the individual’s siblings. Young adults living in the same household with a sibling with depression are at increased risk of emotional distress, reduced social status, diminished self-esteem, and negative stigmatization of their family [8–10, 12]. The objective of this research study was to explore the retrospective experiences of young adults (ages 18–29) who lived with a sibling with depression while they were adolescents.

The participants in the current study reported feeling guilty as a result of their sibling’s behavior and inability to function, as well as anger, confusion, and instability. They described their adolescence as a period of constant tension, anger, and worry. The findings of this study are in line with the findings of previous studies describing the difficulties experienced by siblings living with a brother or sister with a chronic illness or mental health condition [8, 27, 28]. A survey by Incledon [28] found that individuals whose siblings have chronic conditions are at increased risk of developing mental illnesses, such as anxiety and depression. A meta-analysis and review examining how children are affected by the chronic health conditions or emotional disorders of their siblings demonstrated the vulnerability of these children as well as the emotional effects and the impact on the family [29]. The experiences of the participants in the current study resemble those of other family members who provide care for adult relatives with mental illnesses, as documented in previous research [29–32]. A review of 17 studies investigating the experiences of adolescents caring for family members with mental illness revealed that these teenagers regarded caregiving as a burden that caused them significant stress and negatively affected their quality of life and mental well-being. Furthermore, they experienced feelings of social isolation and a sense of being different from their peers of the same age [33]. Studies indicate that family members experience a sense of helplessness and a need for constant vigilance as a result of their lack of knowledge about the disease [34, 35].

Some of the participants in the current study reported that despite the difficult situation at home, they felt they must help the family, even if it meant relinquishing their own needs. Other participants, in contrast, chose to stay away and avoid the stormy atmosphere at home. Indeed, the participants adopted two main approaches to cope with this complex situation: merging or avoidance. Merging entails enlisting oneself to the benefit of the sibling with depression to the point of self-nullification, while avoidance involves cutting oneself off and distancing oneself from the sibling. The merging pattern helped the participants feel useful and valuable in helping cope with the distressful situation at home, but on the other hand it also had a negative impact on their personal lives.

The findings of the present study demonstrate a consistent pattern reflecting the significance of the term "family burden" in characterizing the adverse impact on individuals who care for family members living with mental illness [36]. Family burden encompasses both subjective and objective consequences. The objective costs of caring for a mentally ill family member include less leisure time and the need to assume significant family responsibilities. The subjective costs of coping with family burden find expression in negative emotional states, such as anxiety, sadness, and feelings of embarrassment, [37, 38]. Family members often adopt this coping mechanism based on their compassion for the family member with mental illness. Studies show that in cases where one partner is diagnosed with mental illness after the relationship has begun, the other partner may opt to remain and provide care out of a sense of compassion [39]. These family members expressed concern over the consequences of not assuming the caregiving responsibility, fearing that the affected individual would be left isolated, vulnerable, and without treatment [40]. They believed it was their ethical obligation to adjust to the new situation and dedicate themselves to ensuring the individual receives treatment, even at the cost of self-sacrifices and neglecting their own needs. The term “false self” describes a situation in which individuals feel the environment is not available to accept them and their desires, forcing them to adapt themselves to the environment [41]. Those with a false self must constantly “wear a mask” and act in accordance with the desires and dictates of others around them, while at the same time nullifying themselves and their needs.

In contrast, participants in the current study who adopted the avoidance pattern avoided communicating with their sibling with depression and sometimes even distanced themselves from the entire family in order not to be flooded emotionally. Participants who chose to cut themselves off from their sibling with depression expressed a fear that society would assign negative labels to them because of their sibling’s illness. The research literature contains abundant evidence showing that the individual with mental illness is not the only one who must cope with negative stigmas. Rather, a negative label is often affixed to the entire family in what is known as “stigma by association” [42, 43], such that the family is held responsible for the illness of the family member and even considered to be to blame. Family members who internalize and believe in the truth of this stigma report a high degree of psychological distress, low self-esteem, and low sense of self-worth [43]. As the level of this stigma increases, the family members become more distant from one another [43].

In our study, participants indicated that their relationship with their parents changed after their sibling became ill. The sibling’s difficulties made their parents less available to them and resulted in feelings of neglect, disappointment, loss, and pain. These findings are in line with other research evidence showing that parents’ lack of availability due to the illness of one of their children has a negative impact on the other children in the home [8, 28, 31]. A study investigating the connection between the quality of parental relationships and the mental well-being of siblings of children with chronic illnesses identified a negative correlation between the two variables: the stronger and more intimate the parental relationship, the less likely the siblings were to experience psychological distress and mental illness. The study also examined factors that affect the quality of the relationship and found that parental financial stability, overall well-being, and mental health were significant influences [21].

Moreover, the research participants reported having trouble witnessing their parents’ difficulties, which caused them deep pain and sorrow. They invested major efforts to bring their parents some degree of pleasure, to make things easier for them, and to spare them further pain. The research participants noted that they sought to please their parents in an attempt to compensate them. The findings of the current study reinforce previous findings indicating that quality of parental emotional support acts as a mechanism safeguarding the mental health of the siblings of those with mental health issues [21, 28, 44]. According to family systems theories, a child identified as having mental health problems will have an impact on the entire family system and the relationships within that system [45]. In such a system, the siblings often describe having to take on the role of caregiver or third parent to the child with mental health problems, including monitoring, ensuring the child completes daily tasks, preventing the child from acting inappropriately, and covering up for misbehavior [46]. Siblings often viewed these caregiving responsibilities as burdensome, with the potential to lead to feelings of resentment towards the sibling, thus making sibling conflict more prevalent [30, 31].

Some of the features of the research participants’ relationships with their parents are reminiscent of the experience of losing someone close. This type of loss includes profound feelings of extinction, destruction, and loss. The parents and family members of someone with mental illness experience significant losses that engender grief [47]. Such grief may negatively affect family members’ physical and psychological health as well as their relationship with the affected relative [48]. They mourn the permanent loss of their family member even though this person is still with them physically [47]. Another challenge the participants faced was their parents’ demand that they conceal their sibling’s depression. The research participants remained alone with this distressing experience, without any ability to share it with those close to them. A systematic review and meta-synthesis conducted among family caregivers of people with severe mental illness found stigmatizing attitudes toward people with mental illness and their family members [40]. For the family of someone with mental illness, secrecy and concealment serve as safeguards against stigmatizing the individual and the family, thus avoiding harm, separation, humiliation, or pity on the part of those around them [49]. Secrecy makes it possible for the family to lead a “normal” life. Moreover, the “secret” serves as a component that preserves the family, strengthens its defenses against the outside, and creates a sense of shared destiny. Yet the family secret is also accompanied by feelings of shame. Indeed, in the families of people with mental illness, shame shatters the family’s expectations and often the family’s image as an “ideal” family. Shame awakens feelings such as “we are all less worthy now” and arouses a great deal of vulnerability and sensitivity to the question of what will happen if others find out about the illness [43, 49]. Yet as long as the illness remains a secret, neither the person with the illness nor the family can undergo a therapeutic recovery process. Therefore, healing can only begin by revealing the secret so that the family can conduct an open discussion within the family and later outside the family as well [43, 46].

### Strengths and limitations

The strength of this study lies in the fact that it is a qualitative study focusing on participants who lived with a sibling with depression during their adolescence. The interview format allowed for a more detailed and in-depth investigation of the participants’ perceptions and perspectives, thus adding more information to previous findings regarding this phenomenon. This format led to the creation of a homogeneous group of individuals who lived with siblings with depression, in contrast to studies that examined different mental disorders or combined siblings and parents of the affected individual. Another strength lies in the age of the participants (18–29). The period of emerging adulthood has been the focus of very few studies in this context. Another major advantage of the qualitative approach is that the inquiry is broad, allowing the participants to raise issues that matter most to them. The current study sheds light on several important themes, such as the emotional effects experienced by the participants, their coping patterns that range from closeness to avoidance, and their longing for the relationship they had with their parents before their sibling’s illness emerged.

This study also has several limitations. First, due to its qualitative nature, causal relationships could not be determined. Second, the sample comprised a small cohort of siblings in Israel, so that the results cannot be generalized to other population groups. Third, the data emerging from interviews conducted via Zoom may not be as rich as data emerging from face-to-face interviews. Nevertheless, face-to-face interviews were assessed as less beneficial because they required restrictions in the sampling process given the geographical spread of the participants. Fourth, this study focused on the siblings of people with depression. We created a homogeneous group using inclusion and exclusion criteria. At the same time, because the level of the siblings’ depression was not examined in the study, the intensity of the illness may have differed.

Further research is needed to address the potential cultural, ethnic, gender, and age differences in the siblings’ experiences. The long-term experience of the research participants would be a valuable avenue to explore in the future.

### Conclusion and recommendations

The findings of this study shed light on the experiences of adolescents who grew up with a sibling affected by depression. The participants felt confused, helpless, and anxious due to changes at home. Some described witnessing their sibling’s withdrawal, self-inflicted injuries, and turmoil at home. The participants described having to repress their own problems as teenagers and feeling lonely due to the unstable home situation. Young adults who lived with a sibling with depression during their adolescence reported that their sibling’s condition brought about changes in their routine and forced them to adjust their behavior to cope with the new situation. Some adopted merging behaviors and attempted to protect their sibling, while relinquishing their own personal desires. Others adopted avoidance coping patterns marked by evasion and escape and tried to conceal their sibling’s condition from others. After the sibling’s illness emerged, the participants distanced themselves from their parents. They felt their parents were no longer available for them and therefore avoided asking for help or sharing their negative experiences.

The results of this study suggest that improving our understanding of adolescents who grew up with a sibling with depression may result in more positive and supportive relationships, highlighting the need to consider not only the patient with depression but also the siblings who are emotionally and behaviorally affected. Our findings indicate that intervention and policy approaches in educational settings and in the community are crucial in addressing these issues. Specifically, solutions are needed that can reduce the burdens on siblings by providing them more flexible and tolerant support. To build on the insights gained from the current study, future research should adopt a triangulation approach by conducting follow-up studies that examine the perspective of the sibling with depression as well as provide a more comprehensive understanding of their experiences. Additionally, considering various characteristics of the affected sibling and the entire family, such as age, gender, culture, and single parent status, could provide important context for understanding the impact of depression on siblings. Another recommendation for further research is to interview siblings at different ages and use various qualitative methods, such as diaries and videos, to gain a deeper understanding of their experiences. Lastly, gathering both quantitative and qualitative data at the same time may offer a more complete understanding of the impact of depression on siblings’ emotional well-being and coping patterns.
